# The Role of Cardiac Side Population Cells in Cardiac Regeneration

**DOI:** 10.3389/fcell.2016.00102

**Published:** 2016-09-13

**Authors:** Amritha Yellamilli, Jop H. van Berlo

**Affiliations:** ^1^Stem Cell Institute, University of MinnesotaMinneapolis, MN, USA; ^2^Lillehei Heart Institute, University of MinnesotaMinneapolis, MN, USA; ^3^Department of Integrative Biology and Physiology, University of MinnesotaMinneapolis, MN, USA; ^4^Department of Medicine/Cardiology, University of MinnesotaMinneapolis, MN, USA

**Keywords:** stem cells, side population, cardiac regeneration, heart failure, Abcg2

## Abstract

The heart has a limited ability to regenerate. It is important to identify therapeutic strategies that enhance cardiac regeneration in order to replace cardiomyocytes lost during the progression of heart failure. Cardiac progenitor cells are interesting targets for new regenerative therapies because they are self-renewing, multipotent cells located in the heart. Cardiac side population cells (cSPCs), the first cardiac progenitor cells identified in the adult heart, have the ability to differentiate into cardiomyocytes, endothelial cells, smooth muscle cells, and fibroblasts. They become activated in response to cardiac injury and transplantation of cSPCs into the injured heart improves cardiac function. In this review, we will discuss the current literature on the progenitor cell properties and therapeutic potential of cSPCs. This body of work demonstrates the great promise cSPCs hold as targets for new regenerative strategies.

## Introduction

Heart failure remains a pressing healthcare problem because of its increasing prevalence and high rate of morbidity and mortality (Roger et al., 2004; Heidenreich et al., 2013). Loss of cardiomyocytes during the progression of heart failure is a critical problem that limits the efficacy of current therapeutic approaches. Consequently, multiple strategies to replace lost cardiomyocytes with healthy ones are actively being pursued. One innovative strategy is to enhance endogenous cardiac regeneration, the heart's innate ability to generate new cells, by activating residential cardiac cells to proliferate and differentiate into cardiomyocytes.

Cardiac progenitor cells are excellent targets for these therapeutic strategies because they are multipotent, self-renewing cells that reside in the adult heart. Different populations of cardiac progenitor cells have been identified primarily based on their expression of specific cell surface proteins. Recently, we and others employed genetic lineage-tracing models to demonstrate that c-kit^+^ cells, the most extensively studied cardiac progenitor cells, have a limited ability to differentiate into cardiomyocytes *in vivo* under homeostatic conditions (van Berlo et al., 2014; Sultana et al., 2015; Liu et al., 2016). These findings make it that much more important to assess the regenerative capacity of other cardiac progenitor cell populations, which remain promising targets for new heart failure therapies because of their unique properties. First, cardiac progenitor cells are multipotent; they can differentiate into the main types of cells in the heart: cardiomyocytes, endothelial cells, fibroblasts, and smooth muscle cells. Second, cardiac progenitor cells reside in the heart, opening up the possibility to develop targeted therapies that activate these cells *in vivo* circumventing problems like poor engraftment and immune rejection faced by other cellular therapies. Third, cardiac progenitor cells self-renew to maintain a pool of undifferentiated clones in the heart that is ready to be activated in response to specific stimuli. These key properties mean that cardiac progenitor cells are a responsive population of self-renewing cells, residing in the heart, which can differentiate into the main cardiac lineages. For this reason, it is important to critically evaluate the therapeutic potential of cardiac progenitor cells.

Cardiac side population cells (cSPCs) were the first population of cardiac progenitor cells identified in the heart that possess the three key progenitor cell properties discussed above (Hierlihy et al., 2002). Importantly, cSPCs are distinct from c-kit^+^ cells; they do not express the c-kit protein and c-kit^+^ cells do not display the side population phenotype (Pfister et al., 2005; Unno et al., 2012). Furthermore, microarray analysis performed on c-kit^+^ cells and cSPCs demonstrated that they have distinct transcriptional profiles (Dey et al., 2013). In this review, we will discuss research that established the progenitor cell properties of cSPCs and will highlight the remaining gaps in our understanding of cSPCs that need to be addressed in order to determine the therapeutic potential of cSPCs.

## Molecular basis of the side population phenotype

**Figure 1:**
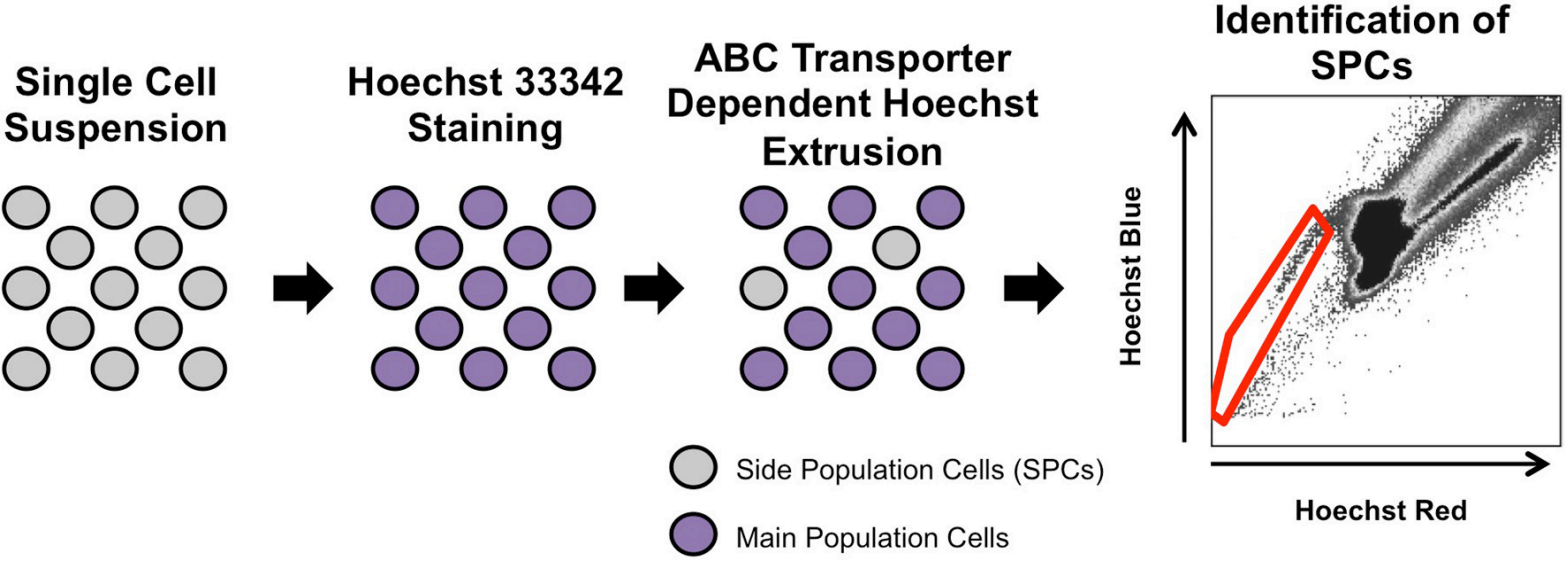
**Isolation of side population cells**. A single cell suspension is isolated and stained with Hoechst 33342. A small percentage of cells are able to extrude Hoechst 33342 out of the cytoplasm through ABC transporters. To identify side population cells, the suspension is analyzed on a flow cytometer (Goodell et al., 1996). The side population cells (red gate) appear to the left side of the main population of cells.

**Figure 2:**
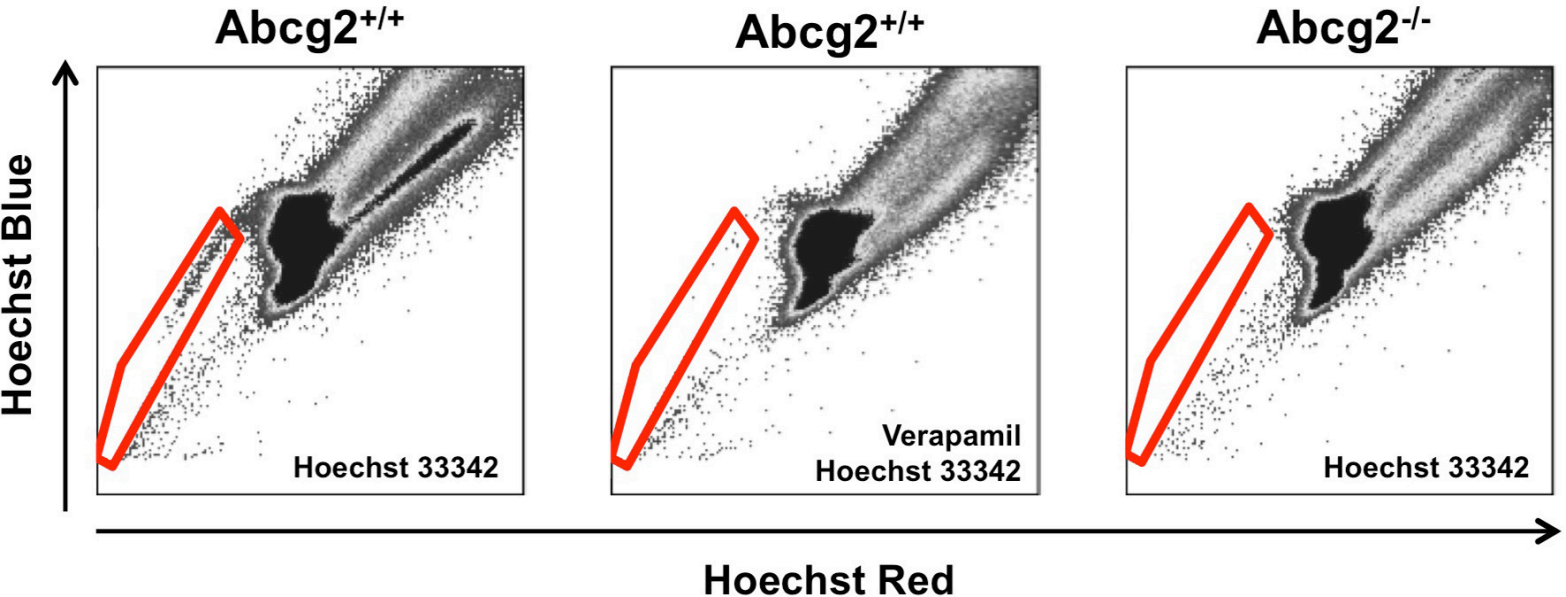
**Flow cytometry analysis of side population cells**. Bone marrow side population cells can be identified by Hoechst 33342 fluorescence (cells within the red gate; Goodell et al., 1996). A sample stained with both Hoechst 33342 and Verapamil, which blocks the side population phenotype, is used to ensure accurate gating and identification of side population cells. In Abcg2 knockout mice, no side population cells are identified (Zhou et al., 2002).

The side population phenotype was first described in 1996, as a way to enrich for hematopoietic stem cells from the bone marrow of adult mice (Goodell et al., 1996). This phenotype identifies cells that have the ability to extrude Hoechst 33342, a cell-permeable, fluorescent, DNA-binding dye, out of the cell through ATP-binding cassette (ABC) superfamily transporters. To isolate side population cells, a single cell suspension from a tissue of interest is incubated with Hoechst 33342, which passively diffuses into the cytoplasm of all cells (Golebiewska et al., 2011). A small number of the stained cells have the ability to extrude Hoechst 33342 out of their cytoplasm. These low-Hoechst staining cells are called side population cells because they appear to the *side* of the high-Hoechst staining cells on a flow cytometry plot (Figure 1). To ensure accurate identification of side population cells, a portion of the Hoechst-stained single cell suspension is also incubated with a chemical that blocks the side population phenotype, such as verapamil (Figure 2; Ambudkar et al., 1999; Montanaro et al., 2004; Sarkadi et al., 2006; Golebiewska et al., 2011).

Since the identification of side population cells in the bone marrow, the side population phenotype has been used to identify stem cells and progenitor cells in tissues throughout the body (Goodell et al., 1996; Jackson et al., 1999; Asakura et al., 2002; Hierlihy et al., 2002; Dekaney et al., 2005; von Furstenberg et al., 2014). In 2002, side population cells were identified in the heart, demonstrating, for the first time, the existence of a pool of residential progenitor cells in the adult heart (Hierlihy et al., 2002). These cells were called cardiac side population cells (cSPCs). Correct identification of cSPCs is dependent on proper digestion of cardiac tissue, accurate cell counts, optimal Hoechst 33342 concentration and precise flow cytometry gating for side population cells (Montanaro et al., 2004). In the adult murine heart, cSPCs account for 0.8–2% of non-cardiomyocytes; however, the number of cSPCs in the heart varies with age (Hierlihy et al., 2002; Martin et al., 2004; Pfister et al., 2005; Oyama et al., 2007; Liang et al., 2010; Noseda et al., 2015). For example, in rats, the number of cSPCs decreases with age, from 4% of non-cardiomyocytes in the fetal heart to 2% in the neonatal heart to 1.2% in the adult heart (Oyama et al., 2007). A similar trend has been observed in humans. In the human fetus, cSPCs account for 1.1% of non-cardiomyocytes; while they account for 0.22% of non-cardiomyocytes in the left atrium of the adult heart (Alfakir et al., 2012; Sandstedt et al., 2012). Surprisingly, this declining trend in numbers of cSPCs reverses later in life, as a recent study demonstrated an increase in cSPCs in aged hearts, noting a 2.3-fold higher number of cSPCs normalized to cardiac mass in 24- to 32-month-old mice compared to 6- to 10-month-old mice (Mulligan et al., 2011).

The side population phenotype is regulated by two ABC superfamily transporters: P-glycoprotein and ABC sub-family G member 2 (Abcg2). Originally, P-glycoprotein and Abcg2 were identified as proteins that confer chemoresistance by extruding drugs out of the cytoplasm of cancer cells (Juliano and Ving, 1976; Doyle and Ross, 2003). They are also responsible for the efflux of xenobiotics, metabolites, and hormones out of cells in the placenta, blood-brain barrier, intestines, kidney, and liver (Schinkel et al., 1994, 1997; Stacy et al., 2013). Of particular interest to this review, P-glycoprotein and Abcg2 are responsible for the side population phenotype in the bone marrow and heart. In bone marrow side population cells, Abcg2 is the exclusive regulator of the side population phenotype (Figure 2; Zhou et al., 2001, 2002; Scharenberg et al., 2002; Jonker et al., 2005). When the genes that encode P-glycoprotein are knocked out, the number of bone marrow side population cells is not reduced compared to wild-type controls (Zhou et al., 2001). Conversely, all bone marrow side population cells are lost when Abcg2 is knocked out (Zhou et al., 2002). Additionally, when Saos-2 cells, HEK-293, or C2C12 cells overexpress Abcg2, they acquire the side population phenotype indicating that Abcg2 is sufficient to confer this phenotype (Scharenberg et al., 2002; Zhou et al., 2002; Martin et al., 2004). In cSPCs, there is an intricate, age-dependent interplay between Abcg2 and P-glycoprotein (Zhou et al., 2002; Pfister et al., 2008). In the neonatal murine heart, Abcg2 is the primary regulator of the side population phenotype. However, as mice age, the role of P-glycoprotein increases until it becomes the primary transporter responsible for the side population phenotype in adult murine cSPCs (Pfister et al., 2008). It is not surprising that both P-glycoprotein and Abcg2 work together to confer the side population phenotype in cSPCs since there is significant overlap between P-glycoprotein and Abcg2 substrates in other cells (Dean et al., 2005; Stacy et al., 2013).

## Progenitor cell properties of cSPCs

### cSPCs are multipotent

They have the ability to differentiate into four major cell types in the heart: cardiomyocytes, endothelial cells, fibroblasts, and smooth muscle cells (Table 1). Several different *in vitro* approaches have been used to differentiate cSPCs into cardiomyocytes (Pfister et al., 2005; Oyama et al., 2007; Yamahara et al., 2008; Lushaj et al., 2012; Belian et al., 2015). When cSPCs are co-cultured with adult rat ventricular cardiomyocytes, they differentiate into cardiomyocytes that are structurally and functionally comparable to adult cardiomyocytes. The cSPC-derived cardiomyocytes electrically couple with surrounding adult rat ventricular cardiomyocytes, express cardiomyocyte specific proteins, and have well-organized sarcomeres. Importantly, their contractility and calcium dynamics when paced are similar to isolated adult cardiomyocytes. Furthermore, fusion between cSPCs and the adult rat ventricular cardiomyocytes was ruled out by co-culturing Green Fluorescent Protein (GFP)-labeled cSPCs with Red Fluorescent Protein (RFP)-labeled adult rat ventricular cardiomyocytes. No double fluorescent cardiomyocytes were observed indicating absence of fusion. Additionally, treatment of cSPCs in culture with Oxytocin or Trichostatin A, without co-culturing with adult cardiomyocytes, was sufficient to generate cSPC-derived cardiomyocytes that express key cardiac proteins, have highly-organized sarcomeres and beat spontaneously.

**Table 1:** *****In vitro*** and ***In vivo*** differentiation methods for cSPCs**.

**Cardiac lineages**	***In vitro* differentiation method**	***In vivo* differentiation method**
Cardiomyocytes	1. Co-culture with adult rat ventricular cardiomyocytes (Pfister et al., 2005)	1. Cryoinjury (Oyama et al., 2007)
	2. Co-culture with neonatal rat ventricular cardiomyocytes (Yamahara et al., 2008)	2. Myocardial Infarction (Liang et al., 2010)
	3. Trichostatin A (Oyama et al., 2007)	
	4. Oxytocin (Oyama et al., 2007; Emmert et al., 2013)	
	5. Dexamethasone (Lushaj et al., 2012)	
	6. MYOCD and TBX5 (Belian et al., 2015)	
Endothelial cells	1. VEGF (Yoon et al., 2007; Lushaj et al., 2012)	1. Cryoinjury (Oyama et al., 2007)
	2. EGF, VEGF, IGF-1, bFGF, Hydrocortisone, and Ascorbic Acid (Liang et al., 2011)	2. Myocardial Infarction (Liang et al., 2010)
		3. Ischemic Limb Injury (Yoon et al., 2007)
Smooth muscle cells	1. VEGF (Lushaj et al., 2012)	1. Cryoinjury (Oyama et al., 2007)
		2. Ischemic Limb Injury (Yoon et al., 2007)
Fibroblasts	–	1. Cryoinjury (Oyama et al., 2007)
**Non-cardiac lineages**	***In vitro***	
Adipocytes	1. MDI-I Mixture (Oyama et al., 2007)	
	2. Adipogenic induction medium (Cambrex Biosciences; (Yamahara et al., 2008))	
Osteocytes	1. β-glycerolphosphate, Dexamethasone, Ascorbic acid-2 Phosphate (Oyama et al., 2007)	
	2. BMP2 (Yamahara et al., 2008)	
Hematopoietic cells	1. Methylcellulose media (Hierlihy et al., 2002)	

*Multiple in vitro methods have been used to differentiate cSPCs into the main cardiac lineages found in the heart, as well as other non-cardiac lineages. In the injured heart, transplanted cSPCs have the ability to differentiate into cardiomyocytes, endothelial cells, fibroblasts, and smooth muscle cells*.

cSPCs also have the ability to differentiate into endothelial cells and smooth muscle cells. When cSPCs are cultured with VEGF or a cocktail of pro-angiogenic factors including VEGF, they begin to express the endothelial markers vWF, CD31, Angiopoietin 1, and Angiopoietin 2 (Yoon et al., 2007; Liang et al., 2010, 2011; Lushaj et al., 2012). After 2 weeks in cell culture, cultured cSPCs form a network of tubular structures (Liang et al., 2011). cSPCs also express the smooth muscle marker, α-smooth muscle actin (α-SMA), when they are cultured with VEGF (Lushaj et al., 2012). When cSPCs were injected into m. quadriceps 1 day after ligation of the left femoral artery, the injected cells engraft, and remain in the tissue for 4 weeks. A small portion of the injected cells expressed endothelial and smooth muscle markers, vWF, and α-SMA, respectively. Consequently, there was increased revascularization and blood flow to the limb, which led to a statistically higher level of limb salvage in the cSPC-injected mice compared to the saline-injected controls (Yoon et al., 2007).

In addition to having the ability to differentiate into cardiac cells, cSPCs can differentiate into extra-cardiac cell types such as osteocytes, adipocytes, and hematopoietic cells. When cSPCs are cultured with BMP2 or a combination of β-glycerolphosphate, Dexamethasone, and Ascorbic Acid-2 Phosphate, they start to express alkaline phosphatase mRNA and protein, a molecular marker used to identify osteoclasts (Oyama et al., 2007; Yamahara et al., 2008). cSPCs can also differentiate into adipocytes when they are cultured in adipogenic media (Oyama et al., 2007; Yamahara et al., 2008). Furthermore, when cSPCs are cultured in methycellulose media, they form granulocyte-macrophage colony forming units that express Mac-1 and Gr-1(Asakura and Rudnicki, 2002; Hierlihy et al., 2002).

### cSPCs reside in the postnatal heart

To determine whether cSPCs are residential cells in the heart and not a population of bone marrow-derived cells that translocate into the heart; newborn mice were lethally irradiated, and then transplanted with GFP-labeled bone marrow. When the mice were 12 weeks old, <1% of cSPCs were labeled with GFP indicating that cSPCs do not arise postnatally from the bone marrow (Mouquet et al., 2005). Furthermore, cSPCs have a distinct expression profile from side population cells isolated from the bone marrow. Cultured cSPCs express important cardiomyocyte transcription factors, GATA4, and MEF2C, as well as cardiomyocyte contractile proteins α-actinin and troponin I. Bone marrow side population cells do not express these proteins under the same culturing conditions. Furthermore, freshly isolated cSPCs do not express CD45 or c-kit, which are both expressed in bone marrow side population cells (Pfister et al., 2005). After injury, however, there is evidence that bone marrow cells can contribute a small percentage to cSPCs and cardiomyocytes (Jackson et al., 2001; Mouquet et al., 2005).

CSPCs are located in specialized niches within the heart. Since the main method of identifying cSPCs utilizes their *ex vivo* phenotype, immunofluorescent staining was performed to identify the precise location of Abcg2^+^CD31^−^ cells in the heart. Ninety-five percent of Abcg2^+^CD31^−^ cells were located in the perivascular area and 5% were located in the interstitial space between cardiomyocytes. There was no difference in the location of Abcg2^+^CD31^−^ cells in the base, middle and apex of the heart. Furthermore, Abcg2^+^CD31^−^ cells expressed both N-cadherin and CD29, which are proteins thought to regulate the interaction of stem cells and progenitor cells with surrounding cells in their niche (Oyama et al., 2007). These data suggest that cSPCs reside in a specific niche, which likely controls their proliferation and differentiation.

While it is clear that cSPCs are residential cells in the postnatal heart, their developmental origins remain unclear. One study identified a potential neural crest origin of cSPCs; while another suggested a cardiac mesodermal origin of cSPCs (Tomita et al., 2005; Noseda et al., 2015). The first study demonstrated that cSPCs have the ability to form spheres in non-adherent culture, called cardiospheres. When cardiospheres, which were generated from all non-cardiomyocytes, not from cSPCs, were implanted into chicken embryos they behaved like cardiac neural crest cells, and homed to the heart. Subsequently, when neural crest cells are lineage-traced they give rise to labeled cardiomyocytes, suggesting that cSPCs are derived from neural crest cells. However, these data provide only inferred evidence that cSPCs are of neural crest origin. The second study used genetic lineage-tracing with multiple Cre lines to identify a cardiac mesodermal origin of cSPCs. Close to 96% of cSPCs were derived from Mesp1-expressing cells, while 50–55% were derived from Nkx2-5- or Isl1-expressing cells (Noseda et al., 2015). Furthermore, 50% of cSPCs were marked as coming from GATA5-expressing cells, suggesting a potential pro-epicardial origin during development. More studies are clearly needed to establish the developmental origins of cSPCs, which could help uncover important pathways regulating the proliferation and differentiation of cSPCs.

### cSPCs self-renew through clonal expansion

When cSPCs were first identified, their clonal properties were demonstrated by their ability to form colonies when co-cultured with neonatal rat ventricular myocytes in methylcellulose media (Hierlihy et al., 2002). Subsequent studies demonstrated that cSPCs can form colonies when cultured in methylcellulose media alone—in the absence of neonatal rat ventricular myocytes (Asakura and Rudnicki, 2002; Martin et al., 2004; Pfister et al., 2005; Liang et al., 2011; Noseda et al., 2015). More importantly, these studies demonstrated that cSPCs formed at least 10 times as many colonies when compared to other non-cardiomyocytes, indicating that cSPCs have the greatest potential for clonal expansion in the heart (Asakura and Rudnicki, 2002; Pfister et al., 2005; Liang et al., 2011; Noseda et al., 2015). To more critically evaluate the self-renewal properties of cSPCs, primary and secondary clones derived from cSPCs cultured in methylcellulose, were propagated and studied for over 10-months. Importantly, these clones retained the side population phenotype, expressed stem cell antigen-1 (Sca-1), and did not undergo replicative senescence (Noseda et al., 2015). Taken together, these *in vitro* studies provide evidence that cSPCs undergo clonal expansion to self-renew their population, an important feature of cardiac progenitor cells.

## Activation of cSPCs

### Abcg2 regulates activation of cSPCs

In addition to regulating the side population phenotype of cSPCs, Abcg2 is a critical regulator of cSPC proliferation, survival, and cardiomyocyte differentiation. Knockout of Abcg2 decreases proliferation of cSPCs in cell culture compared to wild-type cSPCs evidenced by decreased total cell numbers, DNA content, protein content, and Ki-67 and phospho-histone H3 staining (Pfister et al., 2008; Sereti et al., 2013). Abcg2 gene deletion results in upregulation of mRNA expression of cell cycle inhibitors, decreased expression of cell cycle activators, and increased cell cycle duration with more cSPCs in the G_0_/G_1_ phase and fewer cSPCs in S or G_2_/M phases. Additionally, the percentage of cSPCs that undergo asymmetrical cell division increases when Abcg2 is knocked out (Sereti et al., 2013). Consequently, over-expression of Abcg2 increases the total number of cSPCs in cell culture (Pfister et al., 2008). Abcg2 also plays a crucial role in the survival of cSPCs at baseline and in response to oxidative stress (Pfister et al., 2008; Sereti et al., 2013; Maher et al., 2014). When Abcg2 is knocked out, cultured cSPCs undergo a greater level of necrosis and apoptosis under baseline culturing conditions (Pfister et al., 2008). Additionally, Abcg2 knockout cSPCs express higher levels of DNA-damage response genes (Sereti et al., 2013). When these cSPCs are challenged by hydrogen-peroxide treatment, they also have increased cell death compared to wild type cSPCs (Pfister et al., 2008). Finally, Abcg2 regulates the ability of cSPCs to differentiate into cardiomyocytes. While knockout of Abcg2 does not affect cardiomyocyte differentiation of cSPCs, overexpression of Abcg2 decreases the ability of cSPCs to differentiate into cardiomyocytes when they are co-cultured with adult rat ventricular cardiomyocytes (Pfister et al., 2008). Taken together, these studies demonstrate the integral role of Abcg2 in the self-renewal, survival, and cardiomyocyte differentiation of cSPCs. However, the exact mechanisms by which Abcg2 regulates these properties have not been established.

### cSPCs are activated in response to cardiac injury

Since cSPCs are progenitor cells that reside in the heart and have multi-lineage differentiation potential, it is important to determine whether these cells become activated in response to injury. In the murine cryoinjury model of cardiac injury, there was a 3-fold increase in the percentage of cSPCs 3 days after injury. Over the following 2 weeks, the percentage of cSPCs declined but did not return to baseline levels. None of the cSPCs expressed CD45, suggesting that the increase in the percentage of cSPCs was due to proliferation of residential cSPCs and not the result of infiltration of circulating cells that adopt the side population phenotype (Martin et al., 2008). In the more clinically relevant model of experimental myocardial infarction (MI), ligation of the left anterior descending coronary artery led to an acute drop in the number of cSPCs in both the infarct and remote regions of the heart 1 day after injury. Over the next 7 days, the numbers of cSPCs gradually increased back to the number in the sham-operated hearts. The restoration of the numbers of cSPCs after MI was primarily the result of proliferation of residential cSPCs with a small contribution from the bone marrow (Mouquet et al., 2005).

Activation of cSPCs in response to cardiac injury was confirmed in human cardiac tissues. Since Abcg2 is also expressed by endothelial cells in the human heart, cSPCs were identified as Abcg2^+^CD31^−^ cells using immunohistochemical staining (Meissner et al., 2006; Emmert et al., 2013). With this method of identifying cSPCs, the study found an increase in Abcg2^+^CD31^−^ cells in both atria and ventricles of ischemic myocardial samples compared to non-ischemic myocardial samples from patients with other forms of cardiovascular diseases like idiopathic dilated cardiomyopathy or valvular heart disease (Emmert et al., 2013). These findings suggest that myocardial ischemia in the human heart creates a unique environment that enhances the proliferation of cSPCs, similar to cryoinjury in the mouse. Another study demonstrated that therapeutic intervention could also affect the percentage of cSPCs in the human heart. This study utilized Abcg2 staining to identify cSPCs in the setting of chronic heart failure. The number of Abcg2^+^ cells increased in hearts from the time that left ventricular assist devices (LVADs) were implanted to the time the LVADs were removed at the time of cardiac transplantation (Wohlschlaeger et al., 2012). Taken together, these findings indicate that cSPCs in the human heart are responsive to cardiac injury and therapeutic intervention.

To study the *in vivo* response of cSPCs to cardiac injury, an Abgc2-driven, tamoxifen-inducible, lineage-tracing mouse model was used to trace cSPCs *in vivo* after ischemic cardiac injury. In theory, this model would allow the proliferation and differentiation of cSPCs to be identified *in vivo* without using their *ex vivo* phenotype. Unfortunately, initial experiments demonstrated limited labeling efficiency, with only 2% of bone marrow side population cells and differentiated blood lineages derived from bone marrow side population cells labeled (Fatima et al., 2012). More importantly, labeling of cSPCs was not determined (Fatima et al., 2012; Maher et al., 2014; Doyle et al., 2016). Since Abcg2 is the sole regulator of the side population phenotype in bone marrow side population cells and not in cSPCs, it is difficult to imagine that there was robust labeling of cSPCs. Despite these limitations, this model was used to determine the *in vivo* role of cSPCs to cardiac renewal and regeneration. In the embryonic heart, Abcg2-lineage traced cells gave rise to cardiomyocytes, endothelial cells, and vascular smooth muscle cells. After birth, there was a rapid decline in the number of lineage-traced cardiomyocytes, but the number of lineage-traced endothelial cells remained the same. One-month after ischemia-reperfusion injury, no lineage-traced cardiomyocytes were observed. These findings suggest that cSPCs contribute cardiomyocytes to the embryonic heart but not to the postnatal heart. However, with the limited labeling of bone marrow side population cells and without knowing the labeling efficiency of cSPCs, it is impossible to conclusively determine the *in vivo* regenerative potential of cSPCs from these studies.

### Specific factors associated with cardiovascular disease regulate cSPC activation

Not much is known about how the environment in the diseased heart affects activation of cSPCs. One factor that can regulate cSPC activation is the stiffness of the myocardium. For skeletal muscle satellite cells, it has been established that substrate elasticity is critical for muscle stem cell self-renewal (Gilbert et al., 2010). After myocardial infarction, the scar area is considerably stiffer than healthy myocardium. It is possible that this increased stiffness impedes the ability of cSPCs to self-renew and regenerate the heart. To test this hypothesis, cSPCs were cultured on substrates with different elasticity. The rate of cSPC proliferation decreased when cultured on stiffer substrates. Moreover, when cSPCs were co-cultured with neonatal rat ventricular cardiomyocytes on stiffer substrates they showed decreased cardiomyocyte differentiation potential (Qiu et al., 2015). These results indicate that the stiffness of the local environment in the injured heart may alter the regenerative potential of cSPCs. Another factor that can influence cSPC function is Urotensin II, a peptide whose circulating levels increase in cardiovascular diseases like heart failure and hypertension (Russell, 2008). The receptor for Urotensin II and the precursor for Urotensin, are expressed at the mRNA and protein level in cSPCs. Treatment of cSPCs with Urotensin II enhanced their proliferation without altering the cardiomyocyte differentiation potential of cSPCs (Gong et al., 2011). Pressure overload injury induced by transverse aortic constriction (TAC) resulted in upregulation of Urotensin II with a subsequent increase in the percentage of cSPCs. This increase in cSPCs was blocked when mice that underwent TAC surgery were treated with Urantide, a Urotensin II antagonist (Chen et al., 2014). Although the overall consequence on cardiac function was not determined, these results indicate that cSPCs proliferate in response to pressure-overload induced upregulation of Urotensin II. Taken together, these findings demonstrate that signals from diseased hearts promote the activation of cSPCs; however, activated cSPCs are clearly insufficient to completely restore the injured myocardium.

## Therapeutic approaches to repair the injured heart using cSPCs

To test the potential therapeutic applicability of cSPCs, as well as their *in vivo* differentiation capacity, cSPCs have been transplanted in the setting of cardiac injury. Three main studies in animal models have been performed (Table 2). Importantly, all experiments showed differentiation of transplanted cSPCs into cardiomyocytes, endothelial cells and smooth muscle cells, indicating their multi-lineage potential in the setting of post-injury transplantation. In the first approach, cSPCs were isolated from neonatal rats and labeled with GFP (Oyama et al., 2007). These GFP-labeled cSPCs were injected into adult male rats via tail vein injections before cryoinjury of the free wall of the left ventricle. Four weeks after cSPC transplantation, GFP-labeled cardiomyocytes, endothelial cells, smooth muscle cells, and fibroblasts were identified in the border zone. This study demonstrated the ability of cSPCs to home to the injured heart and give rise to GFP-labeled cardiac cells. However, the consequence of cSPC transplantation on cardiac function was not determined. The second approach also used fluorescently labeled cSPCs, but these ones were isolated from adult mice. These cSPCs were injected into the border zone immediately after coronary artery ligation in C57Bl/6j female mice. This study further confirmed the multi-lineage potential of cSPCs upon transplantation, but also did not assess whether transplantation improved cardiac function (Liang et al., 2010). The third study used clonally derived, fluorescently labeled cSPCs isolated from adult mice for transplantation studies. These cSPCs were injected into the border zone immediately after coronary artery ligation in C57Bl/6 adult female mice. Although the majority of transplanted cells did not engraft in the heart, the ones that did engraft differentiated into cardiomyocytes, endothelial cells, and smooth muscle cells. Two weeks after injury, 10% of remaining transplanted cells expressed cardiomyocyte markers troponin I or sarcomeric α-actin, but these cells did not have organized sarcomeres and were mononucleated. Twelve weeks after injury, 50% of transplanted cells expressed cardiomyocyte markers, had organized sarcomeric structures and were binucleated displaying a more mature cardiac morphology. In terms of functional recovery, 1 day after coronary artery ligation, there was no difference in cardiac function between vehicle control and cSPC-derived clones; however, 12 weeks after injury, the hearts injected with cSPC-derived clones showed higher ejection fractions, smaller scar sizes and decreased remodeling (Noseda et al., 2015). Although fusion of injected cells with existing cells was not entirely ruled out as a possible source of labeling, these results suggest that the transplanted cSPCs differentiate into cardiomyocytes that can contribute to cardiac regeneration and improve cardiac function after myocardial infarction.

**Table 2:** **cSPC Transplantation Studies**.

	**Oyama et al., 2007**	**Liang et al., 2010**	**Noseda et al., 2015**
Injury model	Cryoinjury	Coronary artery ligation	Coronary artery ligation
Recipient	Adult male Wistar rats	Adult female C57Bl/6 mice	Adult female C57Bl/6 mice
Donor	Neonatal GFP-transgenic, syngeneic rats	Adult female C57Bl/6 mice	Adult male C57Bl/6 mice
Transplanted cells	300,000 cSPCs	150,000 cSPCs labeled with fluorescent dye	250,000 cSPC clones labeled with lentiviral mOrange
Administration route	Tail-vein injection	Intracardiac injection into border zone	Intracardiac injection into border zone
Functional outcomes	Not assessed	Not assessed	↓ Infarct size
			↓ Remodeling
			↑ Ejection fraction

*Three different approaches have used cSPC transplantation to assess the regenerative ability of cSPCs*.

A common problem with cell transplantation studies in the heart is poor long-term engraftment; this is a problem also faced by cSPC. To overcome the poor engraftment of transplanted cSPCs, biomedical engineering approaches are actively being pursued to develop delivery methods that enhance the viability and engraftment of cSPCs in the heart. One method involved culturing cSPCs in specialized microwells to promote the formation of cSPC aggregates of reproducible sizes (Bauer et al., 2012). The underlying hypothesis was that engrafting aggregates of cSPCs in the heart would enhance cSPC survival by mimicking the cell-cell contact experienced in the stem cell niche. These cSPC aggregates decreased the amount of cell death that occurred in response to oxidative and reoxygenation injury *in vitro*. When these aggregates were transplanted into the heart after ischemic injury, there was an increased retention rate of cSPCs compared to controls where the same number of cells was injected in a single cells suspension. The effect of the implanted aggregates on cardiac function was not evaluated. A second approach involved seeding cSPCs onto a methacrylated gelatin core followed by covering the cells with a silica hydrogel layer (Cha et al., 2014). While the performance of these constructs was not evaluated, they did demonstrate the cSPCs remained viable, proliferated, and were able to spread out off of the gelatin core onto the surrounding surface. Whether these constructs improved transplantation rates of cSPCs or cardiac function after injury was not assessed. These studies demonstrate that cSPCs can be used in combination with biomedical engineering approaches to improve the engraftment rates and potentially the regenerative capacity of cSPCs.

## Conclusion

Overall, these studies are very encouraging because they demonstrate the regenerative properties of cSPCs. They consistently show that cSPCs can be isolated from the heart and can differentiate into the main cardiac lineages. Furthermore, cSPCs are activated in response to cardiac injury and after transplantation into injured hearts. Taken together, these studies provide evidence that cSPCs could be promising targets for regenerative therapies. However, to unequivocally determine the *in vivo* regenerative potential of cSPCs, genetic lineage-tracing studies need to be repeated with a unique cSPC marker. Finally, if it is demonstrated that cSPCs display progenitor cell properties *in vivo*, human studies will need to be performed to assess the regenerative potential of cSPCs in patients.

## Author contributions

AY and JvB wrote the manuscript.

## Funding

This work was supported by grants from the National Institutes of Health (HL112852 and HL130072 to JvB).

### Conflict of interest statement

The authors declare that the research was conducted in the absence of any commercial or financial relationships that could be construed as a potential conflict of interest.
